# A Collection of Cases of Apoplexy, with an Explanatory Introduction

**Published:** 1845-07

**Authors:** 


### Art. XIX.

*A Collection of Cases of Apoplexy, with an explanatory Introduction.* By Edward Copeman, Surgeon.—*London,* 1845. 8vo, pp. 206.

The plan of this little work is excellent; the manner in which the materials are disposed, is highly judicious; and the whole character of the production is indicative of sound judgment, truthfulness, candour, and great modesty on the part of the author. The preface, the whole of which we here transcribe, clearly points out the nature and object of the publication:

"The following collection of cases [250 in number] is published with the view of furnishing sufficient data for determining the comparative merits of different modes of treating apoplexy, and for judging of the expediency of resorting to bleeding for the cure of that disease. It has long been my opinion that the popular, as well as professional, prejudice in favour of bleeding in affections of the brain is not justified by the result of the practice; and in order to convince myself, I collected the following cases. They are transcribed from various books and journals; a few are from my own case book; and I have purposely avoided introducing any author's remarks or comments, that each person who examines them may form an unbiassed opinion. I have arranged in a tabular form for easy reference, the liability of sex and age, the mortality of the disease, the effect of treatment, &c., and have also, not without hesitation, and with a sense of my unfitness for the task, embodied my own views of the subject in a brief explanatory introduction. For this I have no excuse to plead save my anxiety to discover, and to impress upon others the necessity for endeavouring to find out, a more safe, scientific, and successful treatment of apoplexy." (Preface.)

In the "Introductory Remarks," extending only to sixteen pages, Mr. Copeman gives a neat summary of the statistical results furnished by the cases, which well merits perusal. The following extract exhibits the general conclusions as to treatment. We believe Mr. Copeman will find, on inquiring, that the most eminent physicians in this country have long adopted his views as to the impropriety of bloodletting, as a rule of practice, in cases of apoplexy. This warning, however, is, we fear, yet too much called for in some parts of the kingdom.

"A comparison of the success attending the practice of bleeding in apoplexy, with that where bleeding was not employed, as shown by the following cases, is decidedly in favour of the latter; and should be considered sufficiently correct, from the number of cases reported, to neutralize the far too prevalent idea that bleeding is the *only remedy* to be depended upon in apoplexy. The practice of giving emetics when the attack has succeeded a full meal, has not only been safe but effectual. In cases occurring in old age, brandy and other stimulants have restored animation and removed the apoplexy. Purgatives have always been acknowledged to be of essential service in most cases that have recovered. The application of cold to the head, sinapisms to the lower extremities, warm pediluvia, and vesications, have each in their turn appeared to be useful; and are at all events free from the objection that they can either produce or add to the mischief." (pp. 14-5.)

The following statistics are interesting:

| Males. | Females. | Total. | Cured. | Relieved. | Died. |
|---|---|---|---|---|---|
| 170 | 80 | 250 | 68 | 7 | 175 |

| | |
|---|---|
| Proportion of males to females . . . . . . | $2\frac{1}{8}$ to 1 |
| Proportion of deaths to cases . . . . . . | 1 in $1\frac{3}{7}$ |
| Proportion of deaths to recoveries, including those relieved | $2\frac{1}{3}$ to 1 |

| | | |
|---|---|---|
| No. not bled, 26. | Cured, 18; | died, 8 |
| No. bled 129. | „ 51; | „ 78 |

| | |
|---|---|
| No. of cases in which the treatment is specified . . | 155 |
| Proportion of cures in cases treated by bleeding . . | 1 in $2\frac{1}{2}$ |
| Proportion of deaths in ditto, about . . . . . | 1 „ $1\frac{2}{3}$ |
| Proportion of cures in cases not bled . . . . | 1 „ $1\frac{1}{2}$ |
| Proportion of deaths in ditto . . . . . . | 1 „ $3\frac{1}{4}$ |

| | No. | Cured. | Died. | Proportion of Cures to Deaths. |
|---|---|---|---|---|
| Temporal artery opened . . . . | 2 | | 2 | |
| Cupping employed . . . . . | 11 | 6 | 5 | |
| Leeching . . . . . . . | 14 | 4 | 10 | 1 to $2\frac{1}{2}$ |
| Bleeding in the foot . . . . . | 17 | 13 | 4 | $3\frac{1}{4}$ „ 1 |
| General and copious bleeding . . | 85 | 28 | 57 | 1 „ 2 |
| | 129 | 51 | 78. | (pp. 15-6.) |

The sole defect in this book is the scantiness of its materials. Instead of giving the results of two hundred and fifty cases, it ought to have given the results of twice or thrice as many thousands, which the records of medicine could easily have supplied. The author's residence in the country, no doubt, put it out of his power to obtain such data. But we hope he may yet have opportunities of finding them; and we counsel him not to lose sight of the chance of doing so. His present conclusions, however probable, can only be received as inferences provisionally deduced from a few cases of apoplexy; and not at all as the exposition of the present state of our practice and knowledge respecting the natural history and treatment of this disease.

